# Effectiveness of Camouflaged Syringe on Dental Anxiety and Pain During Maxillary Local Anesthesia in Children: A Randomized Controlled Clinical Trial

**DOI:** 10.1155/ijod/6516788

**Published:** 2026-02-13

**Authors:** Bana Darwish, Mohammad Bashier Al Monaqel, Mohammed N. Al-Shiekh, Mawia Karkoutly

**Affiliations:** ^1^ Department of Pediatric Dentistry, Faculty of Dentistry, Damascus University, Damascus, Syria, damascusuniversity.edu.sy

**Keywords:** behavioral management, camouflaged syringe, dental anxiety, local anesthesia, pain perception, pediatric dentistry

## Abstract

**Background:**

The current study aimed to evaluate the effectiveness of a camouflaged dental syringe in reducing dental anxiety and pain during maxillary infiltration anesthesia in children aged 6–9 years, compared to a conventional syringe.

**Materials and Methods:**

This randomized controlled clinical trial included healthy children aged 6–9 years who attended the Department of Paediatric Dentistry at Damascus University between December 2023 and May 2024. All participants required dental treatment in the maxilla involving buccal and palatal infiltration on one side. Participants were randomly assigned to two groups: Group A received local anesthesia with a conventional syringe using the tell–show–do (TSD) technique, and Group B received anesthesia with a camouflaged syringe using the same TSD approach. Dental anxiety was assessed using the facial image (FI) scale. Dental pain was assessed using the face, legs, activity, cry, consolability (FLACC) behavioral pain scale and changes in pulse rate as a physiological indicator for both pain and anxiety. Primary outcome measures were recorded at baseline (*t*_0_), after the TSD technique (*t*_1_), and after anesthesia procedure (*t*_2_).

**Results:**

A total of 70 children (mean age = 7.34 years) were enrolled. The FI scale scores and pulse rates increased significantly overtime in both groups (*p* < 0.05), indicating heightened anxiety during the procedure. However, the camouflaged syringe group showed significantly lower FI scores at *t*_1_ (*p* = 0.001) and *t*_2_ (*p* = 0.005), indicating reduced anxiety levels compared to the control group. Similarly, the camouflaged syringe group reported a significantly lower FLACC score at *t*_2_ (*p* = 0.021), highlighting less reported pain. However, no significant differences were found in pulse rate across time points.

**Conclusions:**

The use of a camouflaged syringe appears to be an effective strategy for reducing dental anxiety and pain in children undergoing local anesthetic injections in the maxillary arch.

**Trial Registration:** ClinicalTrials.gov identifier: ISRCTN51025476

## 1. Background

Anxiety is considered an emotional state triggered by perceived threats and can occur even without actual stimuli. Fear of the dentist, dental procedures, instruments, smells, and the sight of anxious patients are included under dental anxiety. This physiological situation affects individuals of all ages, especially in children, leading to increased distress in clinical settings [1].

Dental fear and anxiety in children are influenced by multiple factors, which can be classified into three main categories. Personal factors include age, gender, general fearfulness, temperament, and cognitive development. Social factors involve parental dental anxiety, the family’s socioeconomic status, and parental preparation and expectations regarding the child’s behavior during dental visits. Environmental factors pertain to the nature of the visit, previous experiences, and the overall dental clinic setting [2].

Dental fear and anxiety represent major obstacles to accessing dental care, frequently resulting in the avoidance of routine treatment. This avoidance can contribute to the progression of oral diseases, including untreated caries, gingival inflammation, and eventual tooth loss. Consequently, children often present only in emergencies, where procedures tend to be more invasive and painful, further intensifying fear and reinforcing a self‐perpetuating cycle of dental anxiety and avoidance [3, 4].

Needle fear is a major contributor to dental anxiety in children, particularly given the routine use of local anesthesia in pediatric dentistry. Visual exposure to syringes can exacerbate anxiety and increase pain perception, especially in predisposed or previously distressed patients. This fear often originates from general dental anxiety or early negative medical experiences, including frequent childhood vaccinations, which may involve physical restraint and contribute to the development of needle phobia [5, 6]. One promising strategy for managing this fear involves camouflaging the syringe, thereby preventing visual triggers and limiting imaginative fear responses. This technique has been identified as effective in reducing anxiety in pediatric patients [7, 8].

Camouflaging is the act of concealing or disguising an object to make it appear less noticeable. It has been proposed as an effective strategy to reduce the visual and psychological threat posed by dental syringes, particularly in children. A syringe with a friendly, nonthreatening appearance may help alleviate anxiety and serve as a distraction during injection [9]. Kettwich et al. [10] demonstrated that camouflaged syringes decorated with child‐friendly designs significantly reduced fear, anxiety, and distress in pediatric and adult oncology patients. Moreover, Bondarde et al. [11] further reported improved pain control and acceptance of local anesthesia in children aged 10–14 years. In addition, Solanki et al. [12] observed similar reductions in fear, anxiety, and pain in children aged 6–11 years. However, there remains a limited number of studies investigating the effect of using camouflaged dental syringes, specifically those covered with brightly colored, animal‐shaped sleeves such as crocodiles, on dental fear and anxiety, and how this relates to perceived pain in children. Based on the findings of Bagher et al. [13], Vallakatla et al. [14], and Melwani et al. [15], the use of a camouflaged dental syringe is an effective alternative to the traditional syringe for administering local anesthesia to children. Research demonstrates that this approach significantly improves children’s behavior and reduces their levels of pain and anxiety during the procedure. Consequently, the camouflaged syringe is recommended as a beneficial tool in pediatric dentistry to enhance the patient experience. Therefore, the current study aimed to evaluate the effectiveness of a camouflaged dental syringe in reducing dental anxiety and pain during maxillary infiltration anesthesia in children aged 6–9 years, compared to a conventional syringe.

## 2. Materials and Methods

### 2.1. Study Design and Ethical Approval

The present study was a randomized, double‐blinded, randomized controlled trial conducted at the Department of Paediatric Dentistry, Faculty of Dentistry, Damascus University, between December 2023 and May 2024. The study adhered to the CONSORT checklist guidelines [16] and complied with the principles outlined in the Declaration of Helsinki for research involving human participants [17]. Ethical approval was obtained from the Ethics Committee of Damascus University (Approval Number 2793/2023). No participant was excluded based on gender, socioeconomic status, or ethnicity. All procedures were clearly explained to the children’s parents or legal guardians before obtaining written informed consent.

### 2.2. Sample Size

The sample size was calculated using 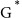
Power software version 3.1.9.4 (Heinrich Heine University, Düsseldorf, Germany). A total of 70 children was deemed sufficient to detect a small effect size (*f* = 0.36) with a statistical power of 80% (1 − *β*) and a significance level of 0.05 [18, 19]. A pilot study involving 10 children was conducted to estimate the effect size [20].

### 2.3. Inclusion and Exclusion Criteria

Eligible participants were healthy children aged 6–9 years requiring dental treatment involving maxillary infiltration local anesthesia. Included participants had no previous dental experience, had not received any sedative or analgesic medication within 3 h before the appointment, and demonstrated positive and definitely positive behavior according to the Frankl Behavior Rating Scale. The children’s behavior was classified according to the Frankl Behavior Rating Scale during the initial screening phase, before randomization and the experimental procedure. Despite having no previous dental experience, their behavior was determined based on their immediate reaction to the new dental environment and interaction with the clinic staff. This initial assessment was used as a screening criterion to enroll only those children who exhibited “positive” or “definitely positive” behavior, ensuring a homogeneous and cooperative study sample [13]. It was assessed by two blinded outcome assessors, with Cohen’s Kappa coefficient values indicating intra‐examiner and inter‐examiner reliability of > 0.8.

Exclusion criteria included children unable to communicate effectively, those with physical or cognitive disabilities, uncooperative children exhibiting disruptive behavior, and those with acute pulpitis or severe dental abscess. Children whose legal guardians refused to provide consent were also excluded.

A total of 76 children referred to the Department of Pediatric Dentistry at Damascus University were initially assessed for eligibility according to predefined inclusion and exclusion criteria. Of these, 70 children met the criteria and were enrolled. They were then randomly assigned to one of two study groups. It was assessed by two blinded outcome assessors, with Cohen’s Kappa coefficient values indicating intra‐examiner and inter‐examiner reliability of > 0.8.

### 2.4. Randomization and Blinding

Following parental consent, the 70 participants were randomly allocated to the study groups using the website https://www.randomizer.org. Each participant was assigned a unique random number. Randomization was performed using block randomization via https://www.randomization.com, allocating participants equally into two randomly permuted groups of 35 children each, with a 1:1 allocation ratio.

To prevent allocation bias, the randomization sequence was generated before the trial by a third person. The investigator conducting the procedures met the participants for the first time in the treatment room after registration and group assignment.

It was a double‐blinded study where both participants and the data analyst were unaware of the group allocations. The children were not informed about their group assignment or the specific aim of the study.

### 2.5. Study Groups

**Figure 1:**
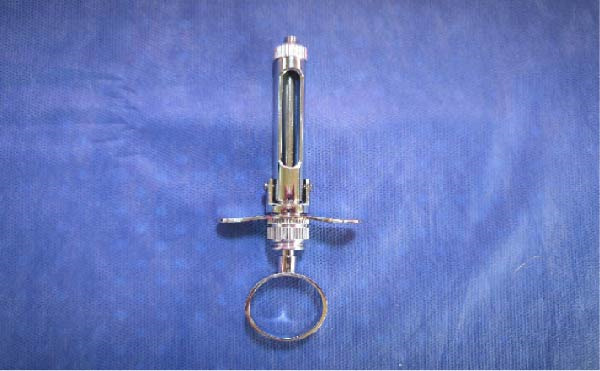
Conventional syringe.

**Figure 2:**
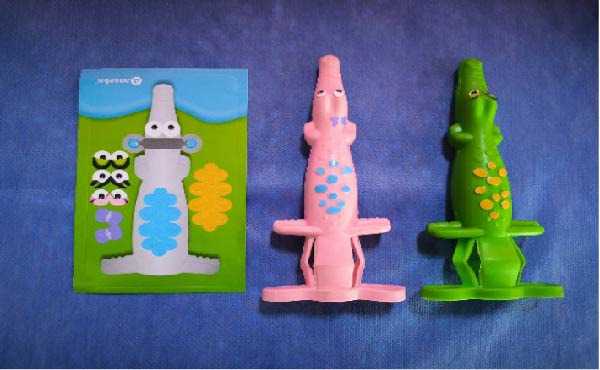
Camouflaged syringe.

Participants were randomly divided into two groups as follows:

• Group A (control group): Received local anesthesia using the conventional syringe (Dental carpule syringe, Dental Laboratorio, China) along with the Tell–Show–Do (TSD) technique (*n* = 35, Figure 1).
• Group B (experimental group): Received local anesthesia using a camouflaged syringe (CROCODILE CASE FOR CARPULE (3u.), Angelus, Londrina, Brazil) along with the Tell‐ Show‐Do technique (*n* = 35, Figure 2).

### 2.6. Primary Outcomes Measure

Primary outcome measures were recorded at baseline (*t*_0_), after TSD technique (*t*_1_), and after anesthesia procedure (*t*_2_).

**Figure 3:**
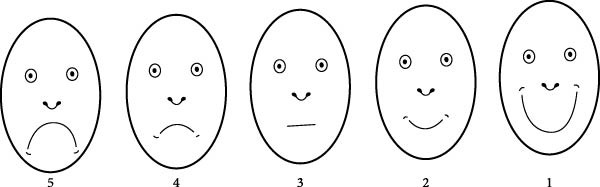
Facial image scale^21^.

Dental anxiety was assessed using the facial image (FI) scale, a self‐reported tool designed to evaluate the degree of fear and anxiety in children. The FI scale comprises five faces ranging from very happy to very sad, corresponding to scores from one to five, where a score of one indicates no anxiety, and a score of five indicates extreme anxiety [21, 22]. The scale was presented to each participant in printed form, and they were asked to select the face that best represented their feelings at the time of assessment. The first FI scale score was recorded while the child was seated comfortably in the dental chair before any intervention (*t*_1_). The second score was recorded following the behavioral management phase using the TSD technique, after the child was introduced to either the camouflaged or conventional syringe based on group allocation, and before the administration of topical anesthesia (*t*_2_). The FI scale is considered a highly valuable tool for assessing dental anxiety in children, particularly due to its simplicity and ease of use among younger age groups [21]. It is widely recognized as one of the most straightforward and effective methods for evaluating pediatric dental anxiety [23, 24] (Figure 3).

In addition, physiological changes in pulse rate (measured beats/min) were recorded at three different time points during the study using a finger pulse oximeter (Alpha, Prolinx GmbH, Düsseldorf, Germany). The first measurement was taken while the child was comfortably seated in the dental chair; the second was obtained after behavioral management using the TSD technique and after the introduction to either the camouflaged or conventional syringe, depending on group assignment. The third measurement was taken 1 min after completion of the local anesthetic injection. Elevated levels of stress, fear, anxiety, and pain are known to increase pulse rate by activating the sympathetic nervous system response [25, 26].

To assess pain perception, children’s behavior during the administration of local anesthesia was video‐recorded using a smartphone camera (Xiaomi 13T, 2023) mounted on the dental chair. Pain was assessed using the face, legs, activity, cry, consolability (FLACC) scale, an observational behavioral pain assessment tool. The FLACC scale evaluates five behavioral domains: facial expression, leg movement, activity, crying, and Consolability. Each domain is scored from zero to two, resulting in a total score ranging from zero to 10, where zero indicates no pain and 10 indicates severe pain [27, 28]. The FLACC scale has been shown to possess high reliability and sensitivity in detecting pain in young children, making it a valid tool in pediatric clinical settings [29].

The primary outcome measures were evaluated by two independent external observers, who were trained pediatric dentistry residents at Damascus University. They were calibrated and trained specifically for this study. Cohen’s Kappa coefficient values indicate intra‐examiner and inter‐examiner reliability of > 0.8.

### 2.7. Intervention

A single dentist, who was both qualified and trained in the relevant methodology, performed all treatments.

The sample was collected from children who were referred to the Department of Paediatric Dentistry at the Faculty of Dentistry, Damascus University. Participation in the study was openly voluntary, and informed consent was obtained. Before initiating the procedure, each child was asked to express their level of dental anxiety upon sitting in the dental chair by selecting a facial expression that best represented their emotional state, using the FI scale. The FI scale was introduced by the dentist.

To record physiological parameters, a pulse oximeter (Alpha, Prolinx GmbH, Düsseldorf, Germany) was placed on the index finger of the left hand. The device was introduced to the child in a child‐friendly manner and described as a happiness meter to enhance acceptance and cooperation.

Behavioral management was carried out using the TSD technique to familiarize the child with the components of either the conventional or camouflaged dental syringe, depending on the assigned group. The explanation was delivered using age‐appropriate and imaginative language. The conventional syringe (Dental carpule syringe, Dental Laboratorio, China) was described as a magic juice cup in the control group. The camouflaged crocodile‐shaped syringe (CROCODILE CASE FOR CARPULE (3u.), Angelus, Londrina, Brazil) was introduced as a brave knight carrying magic juice in the experimental group. Following this behavioral intervention, the pulse rate was measured again, and anxiety was reassessed using the same FI scale.

The injection site was then dried, and 20% benzocaine topical anesthetic (Iolite, Dharma Research Inc., Florida, United States) was applied with a cotton applicator for 1 min. Subsequently, lidocaine 2% with 1:80,000 epinephrine (2% Lidocaine HCL Injection, Huons Co., Ltd, Seongnam, Korea) was administered via infiltration in the maxillary arch. 1 min after the administration of local anesthesia, a third pulse rate measurement was recorded.

Postoperative pain was assessed using the FLACC scale, based on video recordings of the entire procedure. The assessments were conducted by two independent, blinded evaluators who reviewed the recordings. For video recording, a single camera was used, as it provided a sufficient field of view to capture both facial expressions and whole‐body activity. Cohen’s Kappa coefficient values of intra‐examiner and inter‐examiner reliability were > 0.8.

### 2.8. Statistical Analysis

All statistical analyses were performed using IBM SPSS Statistics version 25 (IBM Corp., New York, USA). For categorical variables, descriptive statistics were calculated in the form of frequencies and percentages. For continuous and ordinal variables, descriptive measures included mean, standard deviation, median, minimum, and maximum values. The Kolmogorov–Smirnov test was used to assess the normality of distribution for the continuous variables. In addition, to ensure the homogeneity of the study groups, the chi‐square test was applied to assess gender distribution, while the Mann–Whitney *U* test was used to evaluate age differences. As the quantitative data did not follow a normal distribution, nonparametric tests were applied, including the Mann–Whitney *U* test for comparing values across groups. The level of statistical significance was set at *p* < 0.05.

## 3. Results

**Figure 4:**
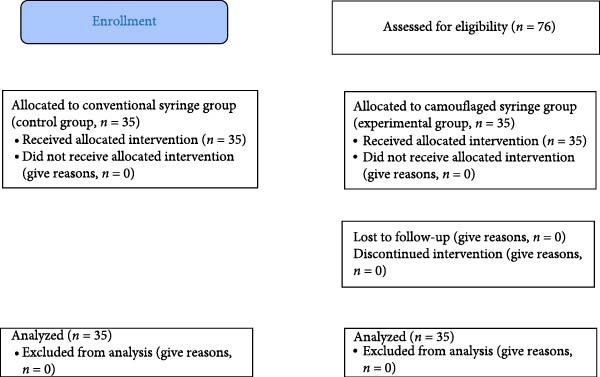
CONSORT diagram.

The CONSORT flow diagram is presented in Figure 4. A total of 70 children, 25 (35.7%) were males and 45 (64.3%) females. The mean age of participants was 7.34 ± 1.089. The FI Scale showed a gradual increase from baseline (*t*_0_) (1.41) to postanesthesia (*t*_2_) (2.04). Similarly, pulse rate increased progressively across *t*_0_ (100.30 ± 11.442), *t*_1_ (105.34 ± 14.436), and *t*_2_ (113.09 ± 15.611). Furthermore, the FLACC scale, assessed only at *t*_2_, had a mean of 3.57 ± 2.082 (Table 1).

**Table 1:** Minimum, maximum, mean, standard deviation, and median values for age, FI scale, pulse rate, and FLACC score.

Variables		Minimum	Maximum	Mean	Standard deviation	Median
Age (years)		6.00	9.00	7.34	1.089	7.00

FI scale	*t*_0_	1.00	3.00	1.41	0.732	1.00
	*t*_1_	1.00	5.00	1.54	0.846	1.00
	*t*_2_	1.00	4.00	2.04	0.939	2.00

Pulse rate	*t*_0_	76.00	130.00	100.30	11.442	100.00
	*t*_1_	77.00	139.00	105.34	14.436	103.50
	*t*_2_	84.00	148.00	113.09	15.611	114.00

FLACC	*t*_2_	0.00	8.00	3.57	2.082	4.00

*Note: t*_0_, at baseline; *t*_1_, after tell–show–do (TSD) technique; *t*_2_, after anesthesia procedure.

Abbreviations: FI, facial image; FLACC, face, legs, activity, cry, consolability.

The Kolmogorov–Smirnov test indicated that age was not normally distributed in the sample (*p* < 0.05). The mean age in Group A was 7.54 years (median = 8.00), while in Group B it was 7.14 years (median = 7.00). The difference in age distribution between the two groups was not statistically significant (*p* = 0.132), suggesting that the groups were homogeneous with respect to age (Table 2).

**Table 2:** Comparison of age between Group A and Group B using the Mann–Whitney *U* test.

Variable	Group A		Group B		*p*‐Value
	Mean ± SD	Median	Mean ± SD	Median	
Age (years)	7.54 ± 1.120	8.00	7.14 ± 1.033	7.00	0.132

*Note:* Group A, control group; Group B, experimental group.

Abbreviation: SD, standard deviation.

In Group A (control group), 15.7% of participants were male and 34.3% were female, while in Group B (experimental group), 20.0% were male and 30.0% were female. The difference in gender distribution between the two groups was not statistically significant (*p* = 0.309) according to Fisher’s exact test (Table 3).

**Table 3:** Frequency and percentage of male and female participants in Group A and Group B using Fisher’s exact test.

Gender		Male		Female		*p*‐Value
		*N*	%	*N*	%	
Studied groups	Group A	11	15.7	24	34.3	0.309
	Group B	14	20.0	21	30.0	

*Note*: Group A, control group; Group B, experimental group; *N*, frequency; %, percentage

The Kolmogorov–Smirnov test indicated that the FI scale, pulse rate, and FLACC scale were not normally distributed in the sample (*p* < 0.05).

Table 4 presents a comparison of primary outcome measures between Group A and Group B at three time points: baseline (*t*_0_), after the TSD technique (*t*_1_), and after the anesthesia procedure (*t*_2_), using the Mann–Whitney *U* test. No significant difference was observed in the FI scale scores between the groups at baseline (*p* = 0.593). However, significant differences emerged at *t*_1_ and *t*_2_ (*p* = 0.001 and *p* = 0.005, respectively), with Group A demonstrating higher FI scores, suggesting greater anxiety or discomfort during and after the intervention (Table 4). Pulse rate comparisons showed no statistically significant differences between groups at any time point (*p* > 0.05, Table 4). Additionally, the FLACC scores, assessed at *t*_2_, were significantly higher in the control Group (A) compared to the experimental Group (B) (*p* = 0.021), indicating increased pain in the control group following anesthesia (Table 4).

**Table 4:** Mann–Whitney *U* test comparing FI scale, pulse rate, and FLACC scores between Group A and Group B.

Variables	Time point	Group A		Group B		*p*‐Value
		Mean ± SD	Median	Mean ± SD	Median	
FI scale	*t*_0_	1.40 ± 0.775	1.00	1.43 ± 0.689	1.00	0.593
	*t*_1_	2.00 ± 0.970	2.00	1.09 ± 0.284	1.00	0.001 ^∗^
	*t*_2_	2.37 ± 0.973	2.00	1.71 ± 0.789	2.00	0.005 ^∗^

Pulse rate	*t*_0_	100.54 ± 9.519	100.00	100.06 ± 13.226	104.00	0.986
	*t*_1_	107.34 ± 12.255	105.00	104.14 ± 16.355	101.00	0.293
	*t*_2_	114.29 ± 15.081	113.00	111.89 ± 16.253	115.00	0.729

FLACC	*t*_2_	4.14 ± 1.801	5.00	3.00 ± 2.209	3.00	0.021 ^∗^

*Note:* Group A, control group; Group B, experimental group; *t*_0_, at baseline; *t*_1_, after TSD technique; *t*_2_, after anesthesia procedure.

Abbreviations: FI, facial image; FLACC, face, legs, activity, cry, consolability; SD, standard deviation.

^∗^Significant difference.

In the control group, a significant difference was observed at baseline (*t*_0_) in the FI scale scores between males and females (*p* = 0.049), with males showing higher mean scores, suggesting greater initial anxiety or discomfort. No other significant gender differences were noted in FI scale scores at subsequent time points (*t*_1_ and *t*_2_), pulse rate at any time point, or FLACC scores after the anesthesia procedure (*t*_2_, Table 5). In the experimental group, no significant gender differences were found in the FI scale or pulse rate, or FLACC scores at any time point. These findings indicate that gender‐related differences in behavioral and physiological responses were limited and mostly confined to the baseline FI scale in the control group (Table 5).

**Table 5:** Mann–Whitney *U* test comparing primary outcome measures by gender within Group A and Group B.

Group A						
Variables	Time point	Male		Female		*p*‐Value
		Mean ± SD	Median	Mean ± SD	Median	
FI scale	*t*_0_	1.73 ± 0.905	1.00	1.25 ± 0.676	1.00	0.049 ^∗^
	*t*_1_	2.27 ± 1.104	2.00	1.88 ± 0.900	2.00	0.249
	*t*_2_	2.27 ± 0.647	2.00	2.42 ± 1.100	2.00	0.766

Pulse rate	*t*_0_	99.18 ± 6.493	100.00	101.17 ± 10.692	99.50	0.789
	*t*_1_	105.82 ± 13.445	100.00	108.24 ± 11.907	106.00	0.444
	*t*_2_	112.09 ± 17.201	114.00	115.29 ± 14.290	113.00	0.557

FLACC	*t*_2_	4.00 ± 2.144	5.00	4.20 ± 1.667	5.00	0.970

**Group B**						

FI scale	*t*_0_	1.29 ± 0.611	1.00	1.52 ± 0.750	1.00	0.303
	*t*_1_	1.07 ± 0.267	1.00	1.10 ± 0.301	1.00	0.808
	*t*_2_	1.71 ± 0.726	2.00	1.71 ± 0.845	1.00	0.884

Pulse rate	*t*_0_	95.64 ± 10.172	95.00	103.00 ± 14.401	106.00	0.109
	*t*_1_	97.57 ± 12.762	94.00	108.52 ± 17.293	115.00	0.050
	*t*_2_	106.36 ± 15.993	105.50	115.57 ± 15.721	118.00	0.110

FLACC	*t*_2_	3.71 ± 2.544	4.00	2.52 ± 1.860	2.00	0.167

*Note:* Group A, control group; Group B, experimental group;*t*_0_, at baseline; *t*_1_, after TSD technique; *t*_2_, after anesthesia procedure.

Abbreviations: FI, facial image; FLACC, face, legs, activity, cry, consolability; SD, standard deviation.

^∗^Significant difference.

The results of the Friedman test showed significant changes over time in both the FI scale and pulse rate within the control and experimental groups (*p* < 0.001, Table 6). In the control group, the mean FI score increased progressively from baseline (*t*_0_: 1.40 ± 0.775) to after the TSD technique (*t*_1_: 2.00 ± 0.970) and after the anesthesia procedure (*t*_2_: 2.37 ± 0.973). Similarly, the pulse rate increased significantly across the same time points (*t*_0_: 100.54 ± 9.519, *t*_1_: 107.34 ± 12.255, *t*_2_: 114.29 ± 15.081, Table 6). In the experimental group, a different pattern was observed. The FI score decreased after the TSD technique (*t*_1_: 1.09 ± 0.284) compared to baseline (*t*_0_: 1.43 ± 0.698), then increased after the anesthesia procedure (*t*_2_: 1.71 ± 0.789), with a significant overall difference (*p* < 0.001). The pulse rate in this group also showed a significant overall increase from *t*_0_ (100.06 ± 13.226) to *t*_2_ (111.89 ± 16.253, Table 6).

**Table 6:** Within‐group comparisons of FI scale and pulse rate at different time points using the Friedman test in Group A and Group B.

Group A			
Variables	Time point	Mean ± SD	*p*‐Value
FI scale	*t*_0_	1.40 ± 0.775	<0.001 ^∗^
	*t*_1_	2.00 ± 0.970	
	*t*_2_	2.37 ± 0.973	

Pulse rate	*t*_0_	100.54 ± 9.519	<0.001 ^∗^
	*t*_1_	107.34 ± 12.255	
	*t*_2_	114.29 ± 15.081	

**Group B**			

FI scale	*t*_0_	1.43 ± 0.698	<0.001 ^∗^
	*t*_1_	1.09 ± 0.284	
	*t*_2_	1.71 ± 0.789	

Pulse rate	*t*_0_	100.06 ± 13.226	<0.001 ^∗^
	*t*_1_	104.14 ± 16.355	
	*t*_2_	111.89 ± 16.253	

*Note:* Group A, control group; Group B, experimental group; *t*_0_, at baseline; *t*_1_, after TSD technique; *t*_2_, after anesthesia procedure.

Abbreviations: FI, facial image; SD, standard deviation.

^∗^Significant difference.

According to the Wilcoxon test, all pairwise comparisons of the FI scale and pulse rate in the control group were statistically significant (*p* < 0.05). In the experimental group, all FI scale comparisons were significant (*p* < 0.001). For pulse rate, significant differences were found between *t*_0_ and *t*_1_ (*p* = 0.001) and between *t*_1_ and *t*_2_ (*p* < 0.001), while the difference between *t*_0_ and *t*_2_ was not statistically significant (*p* < 0.097, Table 7).

**Table 7:** Wilcoxon signed‐rank test for pairwise comparisons of FI scale and pulse rate across time points in Group A and Group B.

Group A			
Variables	Time point	Compared to the time point	*p*‐Value
FI scale	*t*_0_	*t*_1_	0.002 ^∗^
		*t*_2_	<0.001 ^∗^
	*t*_1_	*t*_2_	0.028 ^∗^

Pulse rate	*t*_0_	*t*_1_	<0.001 ^∗^
		*t*_2_	<0.001 ^∗^
	*t*_1_	*t*_2_	<0.001 ^∗^

**Group B**			

FI scale	*t*_0_	*t*_1_	0.001 ^∗^
		*t*_2_	<0.001 ^∗^
	*t*_1_	*t*_2_	<0.001 ^∗^

Pulse rate	*t*_0_	*t*_1_	0.001 ^∗^
		*t*_2_	0.097
	*t*_1_	*t*_2_	<0.001 ^∗^

*Note:* Group A, control group; Group B, experimental group; *t*_0_, at baseline; *t*_1_, after TSD technique; *t*_2_, after anesthesia procedure.

Abbreviations: FI, facial image; SD, standard deviation.

^∗^Significant difference.

## 4. Discussion

Patients’ fear of pain in the dental setting is frequently associated with the administration of local anesthesia. Considerable anxiety could be provoked during the process of injection, along with needle‐related fear. This anxiety is particularly pronounced in individuals with preexisting dental fear and is often linked to avoidance behaviors, such as missed or postponed dental appointments [30].

A range of sensory factors in the dental clinic, such as the visual presence of instruments, auditory stimuli, unpleasant odors, and the anticipation of pain, are recognized as significant triggers of dental fear and anxiety, particularly in pediatric patients [31]. Among these, the sight and sensation of the anesthesia needle and the multisensory experience of the dental handpiece have been identified as particularly anxiety‐inducing [32]. Given the strong influence of visual stimuli on both anxiety levels and pain perception, minimizing such stimuli within the clinical environment may effectively reduce dental fear and enhance children’s behavioral responses during treatment [13, 33].

The dentist holds a pivotal role in managing dental anxiety and optimizing the overall patient experience [34]. Various behavioral management techniques have been introduced to mitigate dental fear and anxiety related to intraoral local anesthesia and the use of the dental syringe, with syringe camouflage being one such approach. Therefore, the present study aimed to assess the effectiveness of a syringe camouflage technique in reducing dental pain and anxiety during maxillary infiltration anesthesia in children aged 6–9 years, compared to a conventional syringe.

The current study included 70 children aged 6–9 years, an age group in which children have the cognitive ability to understand concepts of pain and anxiety, thus improving the validity of self‐reported assessments [35]. Moreover, children at this developmental stage, commonly referred to as school‐aged, are documented to experience heightened levels of dental anxiety [36]. Children with no previous dental experience were involved in this study to eliminate the confounding effect of classical conditioning, which Ranchman’s theory identifies as a central mechanism in the development of dental anxiety. Classical conditioning occurs when a neutral stimulus, such as the dentist or dental instruments, becomes linked to painful experiences during dental treatment, resulting in anxiety responses triggered by these stimuli in subsequent visits [37, 38].

According to the FI scale, this study identified statistically significant differences between the conventional and camouflaged syringe groups after applying the TSD technique. Furthermore, significant differences were also observed between the groups after the post‐ anesthesia procedure. Children in the camouflaged syringe group reported mean FI scores of 1.09 ± 0.284 after applying the TSD technique and 1.71 ± 0.789 after the postanesthesia procedure, whereas children in the conventional group reported higher scores of 2.00 ± 0.970 after applying the TSD technique and 2.37 ± 0.973 after the postanesthesia procedure, indicating elevated anxiety levels in the conventional group at both time points. However, no statistically significant differences were found between the two groups in terms of heart rate. Nonetheless, the camouflaged syringe group consistently exhibited lower mean heart rates across all time points (*t*_0_ = 100.06, *t*_1_ = 104.14, and *t*_3_ = 111.89) compared to the conventional group (*t*_0_ = 100.54, *t*_1_ = 107.34, and *t*_3_ = 114.29).

Camouflaging medical instruments with colorful, cartoon‐like designs has been demonstrated to reduce dental fear and anxiety by altering children’s perceptions and mitigating negative associations with conventional devices. Unlike traditional distraction techniques, which redirect attention away from the stimulus, this approach focuses the patient’s attention on the modified instrument itself, potentially acting as a neurophysiological intervention by engaging brain regions not typically associated with stress responses. This targeted engagement may modulate neural pathways involved in threat perception and emotional processing [13–15]. Sathyaprasad et al. [5], Kettwich et al. [10], and Dahlan et al. [39] confirmed the efficacy of such visual modifications in alleviating anxiety and enhancing children’s cooperation during dental procedures. Findings of this study are consistent with previous studies [5, 9, 12] demonstrating a significant reduction in dental fear and anxiety among children through the use of camouflaged syringes, thereby establishing camouflage as an effective intervention for alleviating such distress. Similarly, the outcome in this study aligns with those of a study by Kettwich et al. [10], which reported that camouflaged syringes and intravenous catheters embellished with various shapes and designs significantly reduced aversion, fear, anxiety, and overall stress in pediatric oncology patients undergoing chemotherapy. Moreover, Bondarde et al. [11] observed that insulin needles decorated with cartoon characters were more readily accepted by children and effectively diminished their dental fear and anxiety. Additionally, the findings of the study further support the result of Babaji et al.’s [40] survey, which indicated a preference for camouflaged syringes among children aged six to 10, in contrast to older children (11 to 14 years), who favored conventional syringes, underscoring the role of syringe camouflage in mitigating dental fear and anxiety. Moreover, the results are consistent with Ahmad et al.’s [41] study, which emphasized the efficacy of camouflaged syringes in reducing dental fear and anxiety by facilitating attentional distraction during dental procedures [42].

According to the FLACC scale, a significant difference was observed between the two groups. The camouflaged syringe group reported a mean score of 3.00 ± 2.21, whereas the conventional syringe group recorded a higher mean of 4.14 ± 1.80. These results indicate that the camouflaged syringe group experienced significantly lower pain levels compared to the control group. The lower mean pain scores in the camouflaged syringe group may be attributed to reduced anxiety levels compared to the conventional syringe group. Anxiety is known to decrease pain tolerance by lowering the pain threshold, thereby intensifying pain perception and emotional distress [26, 42, 43]. As noted by the American Psychological Association, the close interaction between the nervous system and anxiety mechanisms contributes to increased pain sensitivity and hyperalgesia [26].

The findings in this study are in agreement with previous studies [5, 9, 12], which demonstrated a reduction in dental pain levels among children when camouflaged syringes were used, attributing this effect to decreased anxiety. Similarly, the results are consistent with those of Bondarde et al.’s [11] study, who reported that camouflaged insulin needles were associated with reduced pain levels. However, the interpretation for this study differs from Bondarde et al.’s [11] study, which attributed the pain reduction to the use of thinner insulin needles that produced a less painful experience. This study attributes the effect primarily to the reduction of anxiety induced by the camouflage technique. Research by Bagher et al. [13], Vallakatla et al. [14], and Melwani et al. [15] confirms that using a camouflaged dental syringe is an effective alternative to the traditional syringe for pediatric local anesthesia. Studies show it significantly improves children’s behavior and reduces their pain and anxiety, making it a highly recommended tool to enhance the patient experience in pediatric dentistry.

Finally, this study has several limitations. First, the treating dentist was of a single gender, which may have influenced children’s responses. Second, the sample was collected from a single healthcare institution, which may limit the generalizability of the findings. Third, all participating children were within the same age group and had no prior dental experience, making it impossible to assess the effects of age or previous dental exposure. Fourth, only children with positive behavior, as determined by the Frankl Behavior Rating Scale, were included; future studies should consider including children with negative or definitely negative behavior.

## 5. Conclusion

Within the limitations of this study, the camouflaged syringe proved effective in reducing dental fear and anxiety associated with maxillary local anesthesia in children aged 6–9 years with no prior dental experience. Moreover, it demonstrated superior efficacy in alleviating pain compared to the conventional syringe. Further studies are warranted to explore the effectiveness of camouflaged syringes across a broader range of pediatric age groups and in children with a history of negative dental experiences.

**Nomenclature**
TSD: Tell–show–do
FI: Facial image
FLACC: Face, legs, activity, cry, consolability.

## Ethics Statement

Ethical approval was obtained from the Local Ethics Committee of Damascus University (Approval Number 2793/2023). Patients legal guardians or parents signed written informed consent before enrollment. The study was conducted according to the CONSORT guidelines and the Declaration of Helsinki.

## Consent

The authors have nothing to report.

## Disclosure

All authors have read and approved the manuscript.

## Conflicts of Interest

The authors declare no conflicts of interest.

## Author Contributions

Bana Darwish research concept and design, collected data, contributed to writing. Mohammad Bashier Al Monaqel research concept and design, supervised the project, performed critical revision of the manuscript. Mohammed N. Al‐Shiekh extracted the data and performed the statistical analysis, contributed to writing. Mawia Karkoutly contributed to writing.

## Funding

This research is funded by the Damascus University (Grant 501100020595).

## Data Availability

The datasets generated during and/or analyzed during the current study are available from the corresponding author upon reasonable request.
